# Multi-scale correlation of impact-induced defects in carbon fiber composites using X-ray scattering and machine learning

**DOI:** 10.1038/s41598-024-76105-6

**Published:** 2024-10-17

**Authors:** Alexander H. Sexton, Heikki Suhonen, Mathias K. Huss-Hansen, Hanna Demchenko, Jakob Kjelstrup-Hansen, Matthias Schwartzkopf, Matti Knaapila

**Affiliations:** 1 Department of Physics, Norwegian University of Science and Technology, 7491 Trondheim, Norway; 2 DPI , P.O. Box 902, 5600 AX Eindhoven, The Netherlands; 3 Department of Physics, University of Helsinki, Helsinki, Finland; 4 Mads Clausen Institute, NanoSYD, University of Southern Denmark, 6400 Sønderborg, Denmark; 5 SDU Climate Cluster, University of Southern Denmark, 5230 Odense, Denmark; 6 Deutsches Elektronen-Synchrotron DESY, 22607 Hamburg, Germany

**Keywords:** Polymers, Scientific data

## Abstract

Impact-induced defects in carbon fiber-reinforced polymers (CFRPs)-spanning from nanometer to macroscopic length scales-can be monitored using an aggregate of X-ray-based methods, but this is impractical in typical field conditions. We report on a low-velocity impacted CFRP, which is mapped using small- and wide-angle X-ray scattering and X-ray computed tomography, and employ machine learning for correlating material parameterizations derived from these techniques. The observed 1 $\mu$m to 1 mm-sized defects are parameterized in terms of relative density and fiber orientation indicative of fiber failures (kink bands), and the nanometer sized defects in terms of crystal size and unit cell frustration. The 30 to 300 nm defects are parameterized by a power-law scattering decay, differentiating fractal-like behaviors. We find three spatial domains experimentally and by K-means Clustering: Domains of severe damage (with a visual dent), intact domains (without visual or measurable defects) and a transition domain (defects measurable by X-rays). How the parameters are correlated and how they overlap between the domains are discussed. All parameters are able to point to the detrimental fiber breakage in the severe damage domain, and scattering decay also in the transition domain, for example. How individual parameters determined from one experimental technique can be predicted from that of another is also described.

## Introduction

Continuous carbon fiber-reinforced polymers (CFRPs) consist of macroscopically aligned fibers within thermoplastic polymer matrices. Due to their high durability and strength-to-weight ratio, these materials are in increasingly high demand for applications within aerospace and automotive, pressure vessels, wind turbines, protective armors as well as for high-end sporting equipment^1,2^. Though, during materials processing or operation, the composites may become damaged, either by overloading, fatigue or various impact events, leading to defect types such as matrix cracking, delamination and fiber failure, which might be detrimental to the integrity of the material^3–6^. These defect hierarchies range from nanometer to macroscopic length scales^7^, and several experimental methods are therefore required for obtaining a complete picture of the material state. Structural health monitoring (SHM)^8^ can be conducted using methods which include ultrasonic scans^9^, acoustic emissions^10^, vibrothermography^11^, surface reflectometry^12^, infrared thermography^13^, terahertz imaging^14^, microwaves^15^, and dielectric response^16^. Typical for these efforts is to seek for complicated relations which ultimately would allow for accurate lifetime prediction of real-world composites of significant structural complexity, and not only model materials. Consequently, this may lead to elaborate datasets and time-consuming analysis, but defect monitoring should at the same time allow for fast and economical quality control in production lines and field conditions etc.

X-ray-based methods are ubiquitous in studies of polymer materials, composites and their defects. For examining defects in composites, much emphasis has been put on X-ray computed tomography (CT), which includes quantifying matrix cracking and inter-ply delamination during shear deformation^17^, studying crack-initiation processes^18^, characterizing void and fiber distribution in 3D-printed composites^19^, predicting void nucleation^20^, and for investigations of defects as a result of compression loads^21^. In describing defects and mechanisms at the molecular and intermolecular levels, small-angle and wide-angle X-ray scattering (SAXS/WAXS) have been employed for investigations of interface structures^22,23^ and fiber orientation^24,25^. Attention has been placed on mapping crystallinity during tensile and fatigue testing^26,27^, as well as on monitoring microcavities^28,29^, unit cell frustration and lattice strains^30,31^. However, the literature directly connecting characterizations across relevant length scales is for carbon fiber composites less comprehensive, even though it has been attempted in other composite materials^32^. Such characterizations by X-rays do not only depend on several instruments, but also large datasets, which means another problem for fast quality control.

Machine learning (ML) methods are commonly employed to find correlations in large amounts of data for SHM of composites and polymers^33,34^. These methods include Support Vector Machines and K-means clustering combined with mechanical measurements^35^, acoustic emissions^36^, dielectric measurements^37^, electron microscopy^38^, to mention a few. Where large datasets are obtained from synchrotron radiation, ML methods are used in pre-processing and real-time analysis^39^. For example, deep learning techniques have been used for visualizing 2D SAXS patterns in low-dimensional latent spaces, allowing for rapidly capturing key features and trends in large datasets, as well as a priori exploration of processing parameter spaces^40^. Similar methods are also in use for classifying crystallographic dimensionality and space-groups from diffraction patterns^41^, and for classifying SAXS data in terms of appropriate models for analysis, alleviating the need for domain experts^42^.

When the combination of X-rays and ML in SHM is concerned, segmentation of tomographic data has been successful^43^, predicting tomograms from data of other SHM methods^44^, as well as using crystallographic data as predictors for defects^45^. We have recently reported similar ideas for continuous CFRPs and demonstrated applicability of WAXS together with ML^46^. Interestingly, we have been able to classify early stage tensile defects that show minor effect that are fully invisible for any macroscopic or visual defect monitoring. This encourages us to proceed with a full X-ray characterization, and defects reaching from early stage to severe stage and from nanoscale to macroscopic length scale.

In this paper, we study a low-velocity impact-tested continuous CFRP with polyamide-4,10 with visual damage. We begin with CT and connect this to a comprehensive SAXS/WAXS mapping of the impact site. We parameterize these data by identifying suitable descriptors at each structural level, and identify different spatial domains. These include domains of severe damage (visual), intact domains (without visual or measurable defects) and a transition domain (without visual damages but with defects measurable by X-rays), segmented by using the K-means clustering method. We present correlations between the parameters and how they relate to each others in the respective domains, and also report on the performance of SVMs for regression, using the derived parameters as input-output pairs. The results demonstrate that it is possible to form a framework where partial X-ray datasets can be used as criteria for the overall materials failure; or where datasets from one length scale can be used as phenomenological indicators for parameters in another length scale. This has implications for fast defect detection in carbon composites and matrix polymers.

## Experimental section

### Materials and mechanical testing

**Fig. 1:**
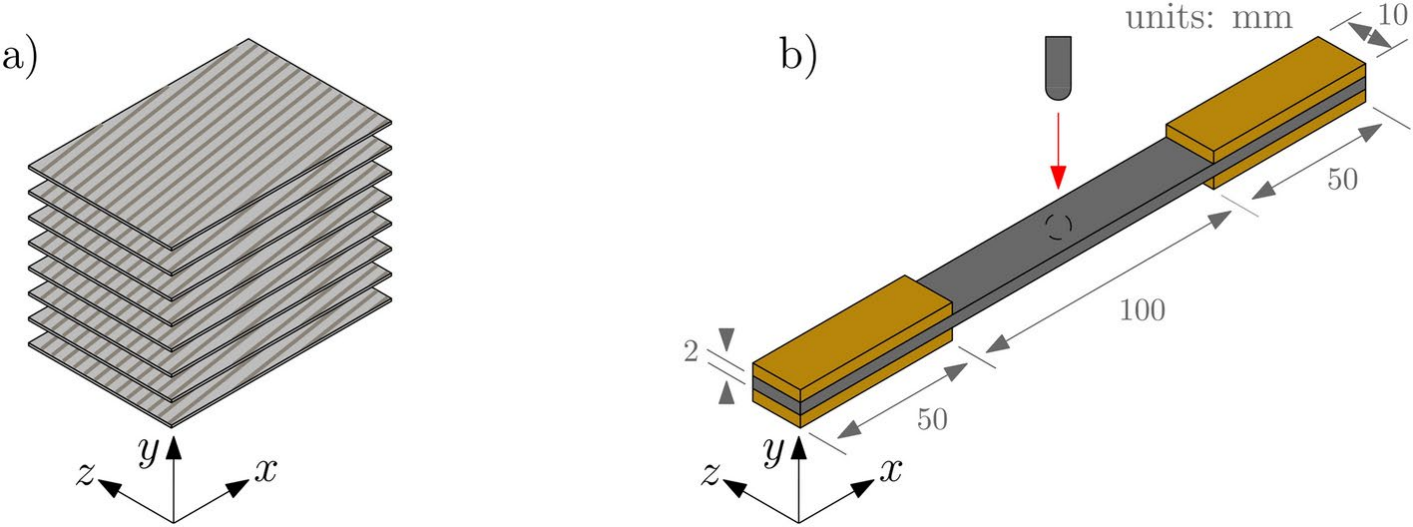
Illustrations of the employed material sample. (**a**) Composite tapes with carbon fibers oriented at $10{^\circ }$, making up the laminated material. (**b**) Dimensions of the sample (with tabs). Defects were introduced by low-velocity impact at the middle of the sample, indicated by the red arrow.

**Fig. 2:**
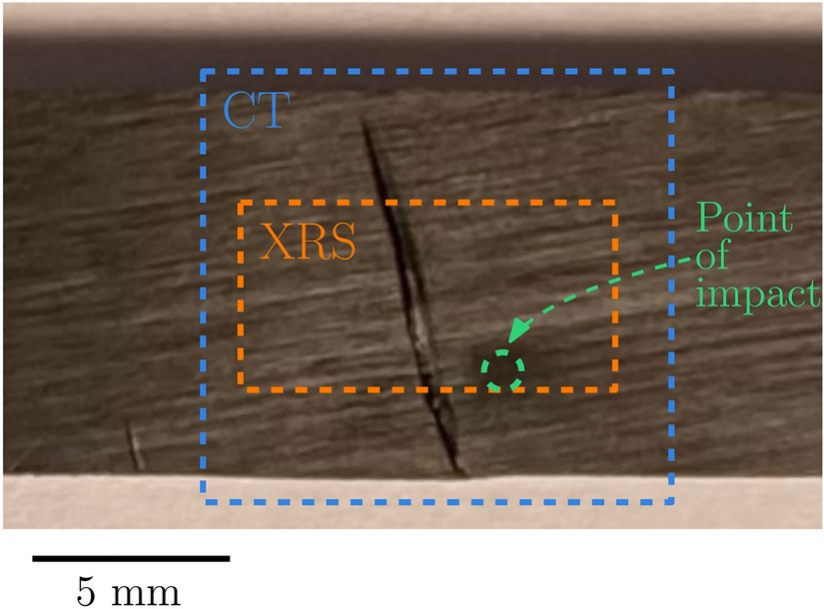
Photo of the studied sample (*x-z* plane) after mechanical tests. Dashed lines show the point of impact (green) and areas mapped by CT (blue) and X-ray scattering (orange).

Fig. 1a-b illustrates the employed sample configuration. Fig. 2 shows the a photo of the sample with apparent impact site, also indicating areas mapped by X-ray microtomography and X-ray scattering. The sample was a continuous carbon fiber-reinforced thermoplastic composite with a polyamide-4,10 matrix and 60 wt. % fiber content (DSM Engineering Materials). Eight layers of tapes in a unidirectional configuration were hot pressed, forming a panel of approx. 2 mm thickness. From this, the sample was cut using waterjet cutting, to form a 200 x 10 $\hbox {mm}^2$ size specimen with fibers oriented at $10{^\circ }$ with respect to the length axis (*x*). To induce barely-visible-damage, the sample was impacted in the middle using CEAST 9350 drop tower. During the test, the sample was supported by a 5 cm thick elastic polyurethane foam, and fixed up against a clamping ring. The drop tower used a 5.78 kg impactor mass with a hemispherical striker tip (20 mm diameter), striking the sample with an impact velocity of 1.17 m/s (4 J impact energy). The impactor was equipped with an 18 kN load cell. Fig. S1 plots the collected force-time history (Supplementary Information).

### X-ray computed tomography

X-ray computed tomography (CT) was done using a Phoenix Nanotom device (Phoenix X-ray Systems + Services GmbH, currently a part of Waygate Technologies owned by Baker Hughes). The sample was placed on the sample stage at 30 mm from the X-ray source, and the detector was placed further 170 mm behind the sample. X-ray voltage of 60 kV and current 150 ${\upmu }$A were used without any filtering of the beam. The number of projection images recorded was 2000 over 360 degree rotation, giving angular step of 0.18 degrees. At each rotation angle a 4 x 500 ms exposure was made. The data was then reconstructed using phoenix datos|x 2 reconstruction software version 2.4.0 (Phoenix X-ray Systems + Services GmbH), giving a total reconstructed volume of 15 x 15 x 15 $\hbox {mm}^{3}$ with a voxel size of 7.5 ${\upmu }$m. A projection of the reconstructed volume is illustrated in Fig.2.

For comparing the reconstructed volume with the spatially mapped x-ray parametrizations (*vide infra*), the tomograms were projected such as to be representative of the average absorption coefficient $\bar{\mu }$ of the sample through the fractured surfaces’ normal. The greyscale value of the tomogram voxels were thus averaged for each position (*x*, *z*) on the sample, as

1 $$\begin{aligned} \bar{\mu }_{xz} = \frac{1}{N_y} \sum _j^{N_y}\mu _{xz, j}, \end{aligned}$$

with $N_y$ the number of voxels containing the composite material in the *y*-direction. This image processing was done using ImageJ software^47^. Furthermore, a parameter representative of the material density (“pseudo-density”) was defined as $\bar{\mu }$ normalized to the observed range (i.e. a value 1 corresponding to the maximum observed (air) and 0 the minimum (polymer)) subtracted from one:

2 $$\begin{aligned} \hat{\rho } = 1 - \frac{\bar{\mu }}{\text {max}(\bar{\mu }) - \text {min}(\bar{\mu })}. \end{aligned}$$

### X-ray scattering

**Fig. 3:**
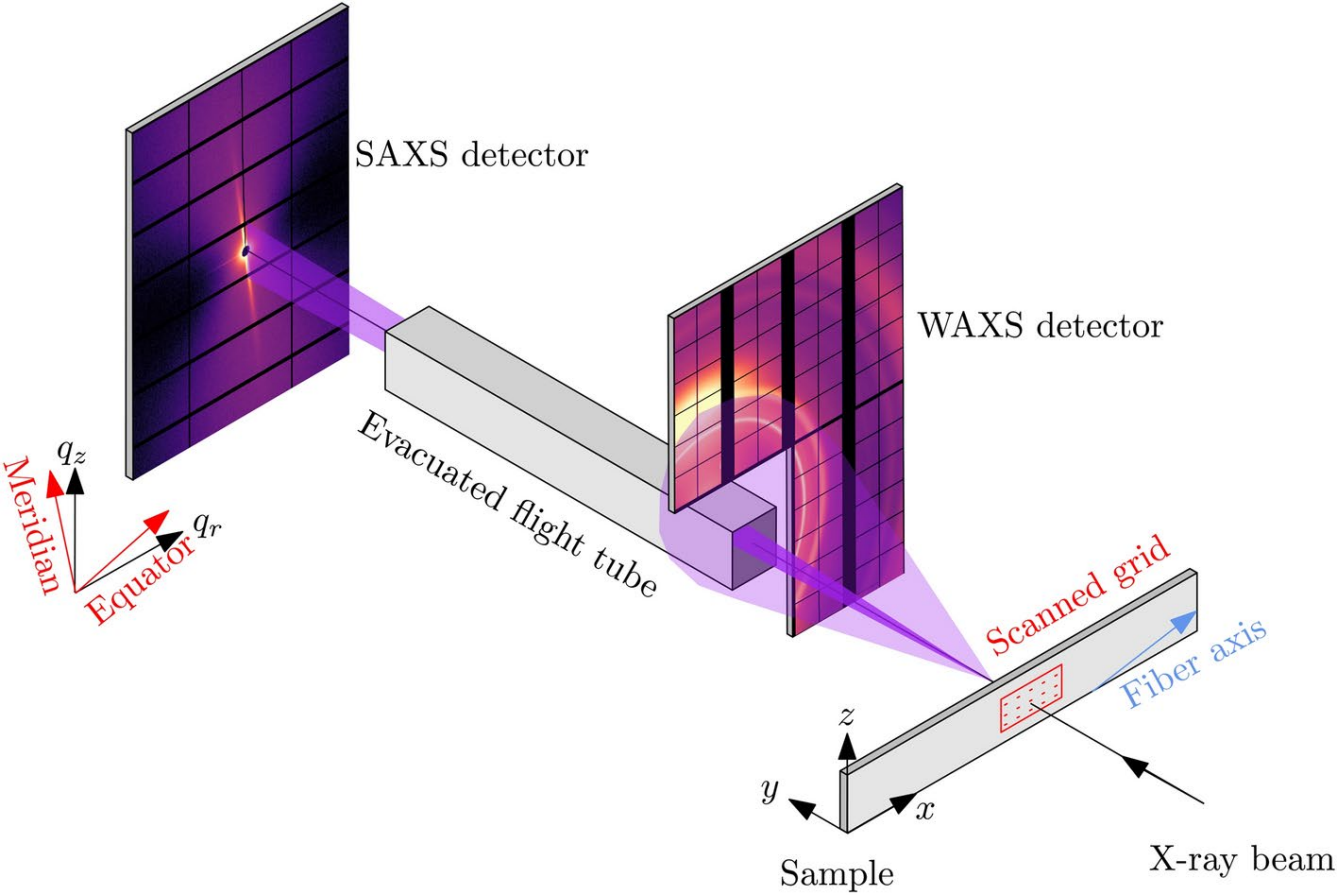
Geometry of the the X-ray scattering experiment.

Fig. 3 illustrates the experimental geometry for SAXS/WAXS experiments. The scattering experiments were conducted at the Micro- and Nanofocus beamline P03 at the Deutsches Elektronen-Synchrotron DESY in Hamburg (Germany)^48^. The energy of the incoming beam was $$E=11.87$$ keV, corresponding to a wavelength of $$\lambda = 1.044$$ Å, and the beam size was $$30 \times 22$$$$\mu \hbox {m}^2$$. The wide-angle scattering was recorded by a LAMBDA 9M (X-Spectrum, 55 $${\upmu }$$m pixel size) detector, whose geometry allowed for the direct beam and small angle scattering to continue downstream, and be simultaneously recorded by a Pilatus 2M (Dectris, 172 $${\upmu }$$m pixel size) detector. The X-ray path downstream of the WAXS detector went through an evacuated flight tube, to minimize parasitic air scattering. The distance from the sample to the WAXS detector was approx. 225 mm, and to that of the SAXS detector approx. 9550 mm. These parameters led to the *q*-ranges 0.025-0.8 $$\hbox {nm}^{-1}$$ and 13.5-19 $$\hbox {nm}^{-1}$$ respectively. The sample was mounted horizontally on a motorized stage, and scanned by 1 second shots in transmission mode, in a grid-like pattern in the region with visible damage. The grid consisted of $$101 \times 51$$ linearly spaced points, with a step size of 100 $$\mu$$m, totalling 5151 X-ray shots. The mapped area is shown in Fig. 2.

**Fig. 4:**
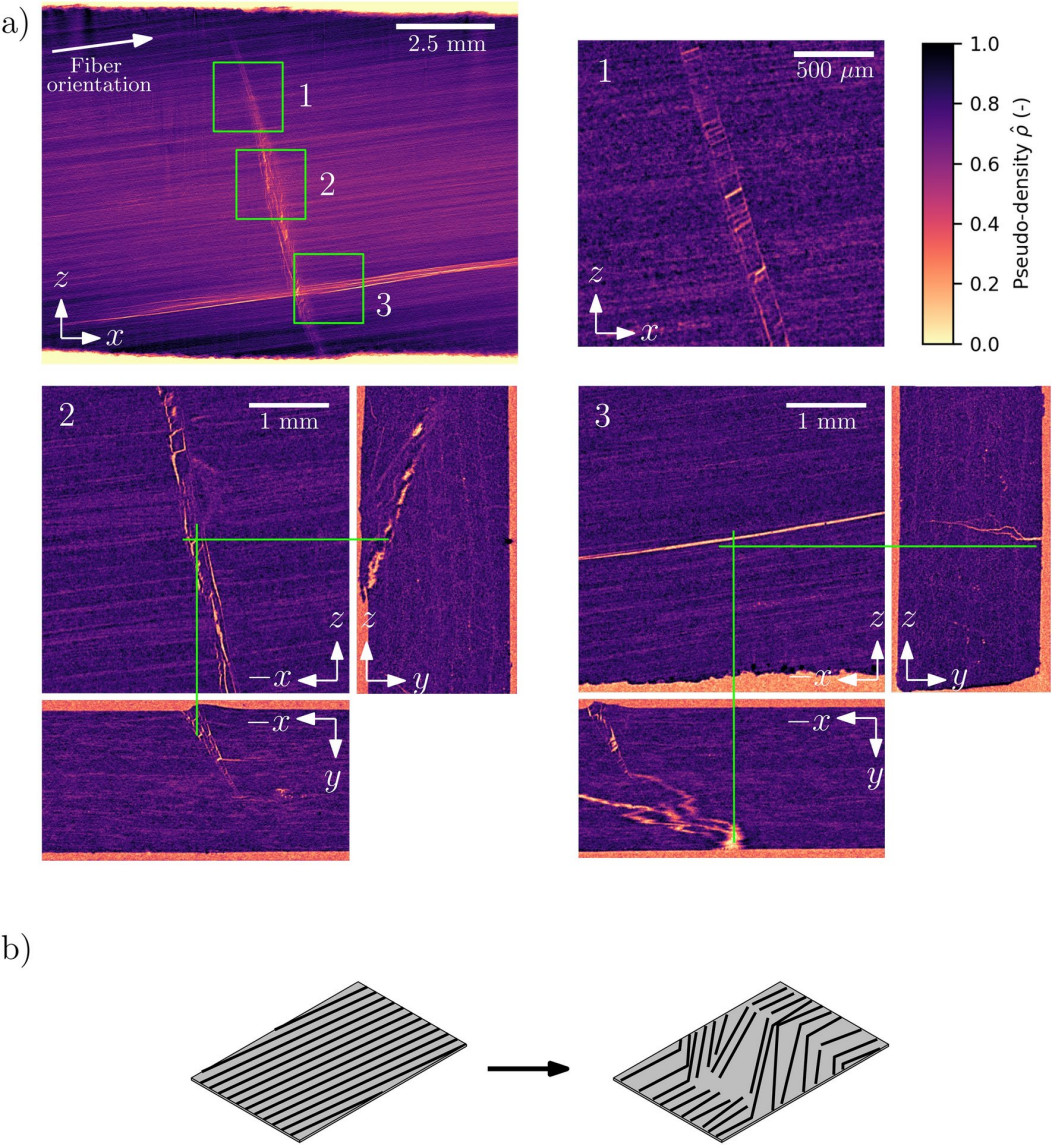
(**a**) CT intensity projection of the (*x*, *z*)-plane of the sample, with selected close-ups of orthogonal cross-sectional slices. 1: Kink bands perpendicular to the fiber orientation. 2-3: Crack structures. White arrows mark the experimental geometry and fiber orientation. The green axes indicate the position of orthogonal slices (e.g. one axis for the *y*-coordinate of the (*x*, *z*)-view and *z*-coordinate of the (*x*, *y*)-view mutually). (**b**) Illustration of the kink band defect.

The SAXS images were azimuthally integrated in two $10^\circ$ wedges along the image equator and meridian (*vide infra*). The meridian axis was defined to follow the azimuthal angle of maximum intensity, i.e. $90^\circ$ off-set from the (local) fiber orientation, and the equator along the fiber orientation. To account for out-of-plane fiber waviness in the sample, this angle was fitted in each scattering image. The resulting (integrated) intensity profiles showed a characteristic decay and an interference maximum, and were therefore fitted using a combination of a power-law function and a Lorentz distribution function

3 $$\begin{aligned} I(q) = A\cdot q^{-\tau } + \frac{B}{\pi }\left[ \frac{\sigma }{(q-q_0)^2 + \sigma } \right] + \text {background}, \end{aligned}$$

where $\tau$ is the power-law exponent and $q_0$ and $\sigma$ are the center and width of the interference maximum respectively. The *A* and *B* are scale factors.

In order to describe the local fiber alignment, SAXS images where also integrated radially, resulting in the intensity as a function of the azimuthal angle $$\chi$$. To minimize artefacts from the detector gaps, the integration was done only in the range $$q\in [0.20, 0.22]$$$$\hbox {nm}^{-1}$$. The obtained profiles were used for characterizing the local fiber alignment, using the $$P_2$$ orientation coefficient:

4 $$\begin{aligned} P_2 = \frac{3\langle \cos ^2(\chi -\chi _0)\rangle -1}{2}. \end{aligned}$$

The reference angle $\chi _0$ is here defined to follow that of the determined meridian. The mean cosine square term (with a zero reference angle) was estimated by

5 $$\begin{aligned} \langle \cos ^2(\chi )\rangle = \frac{\int _0^\pi I(\chi )\cos ^2\chi \sin \chi \textrm{d}\chi }{\int _0^\pi I(\chi )\sin \chi \textrm{d}\chi }, \end{aligned}$$

To account for the possibility of fibers wrinkling/miss-alignement both clockwise and anti-clockwise, Eq. 5 was evaluated using both directions w.r.t. the reference angle, i.e. with integration ranges $[\chi _0 - 90^\circ , \chi _0]$ and $[\chi _0, \chi _0+90^\circ ]$, and the $P_2$ coefficient was thus calculated using the average of the two cosine squared terms, i.e. $\langle \cos ^2(\chi )\rangle _{\text {clockwise}}$ and $\langle \cos ^2(\chi )\rangle _{\text {anti-clockwise}}$.

WAXS images were integrated azimuthally along a ${10}^\circ$ wedge centered around the equator. The equatorial angle of the WAXS signal was defined to be equal to that of the SAXS. The resulting profile, representing the polymer matrix, was deconvoluted into the respective diffraction peaks by performing curve fits. The Bragg reflections and the amorphous phase of the polymer were each represented by Pseudo-Voigt functions and the background from the crystalline phase by a linear function. The curve fitting was done using the lmfit library^49^. The parameters resulting from the curve fits were used to derive two quantities relating to the 100 and 010/110 reflections from the triclinic $\alpha$-phase unit cell^50^. Firstly, the inverse space distance of these Bragg reflections,

6 $$\begin{aligned} \hat{q} = q_{010/110} - q_{100}, \end{aligned}$$

was extracted. Secondly, the Scherrer equation was used to estimate the crystal size:^51^

7 $$\begin{aligned} L_{hkl} = \frac{2K\pi }{\beta _{hkl}}. \end{aligned}$$

Here, *K* is a shape factor (set to 0.9) and $\beta _{hkl}$ is the fitted full-width-half-maximum of the *hkl* reflection. This measure results in a lower bound for the crystal size, due to unaccounted peak broadening arising from the sample thickness, possible strain and paracrystallinity, as discussed for a similar case elsewhere^52^. The instrumental factor in peak broadening was negligible (see Fig. S2 in the Supplementary Materials). The apparent crystal size was further represented as the geometric mean of the respective components along the two reflection planes:

8 $$\begin{aligned} \hat{L} = \sqrt{L_{100}\cdot L_{010/110}}. \end{aligned}$$

**Fig. 5:**
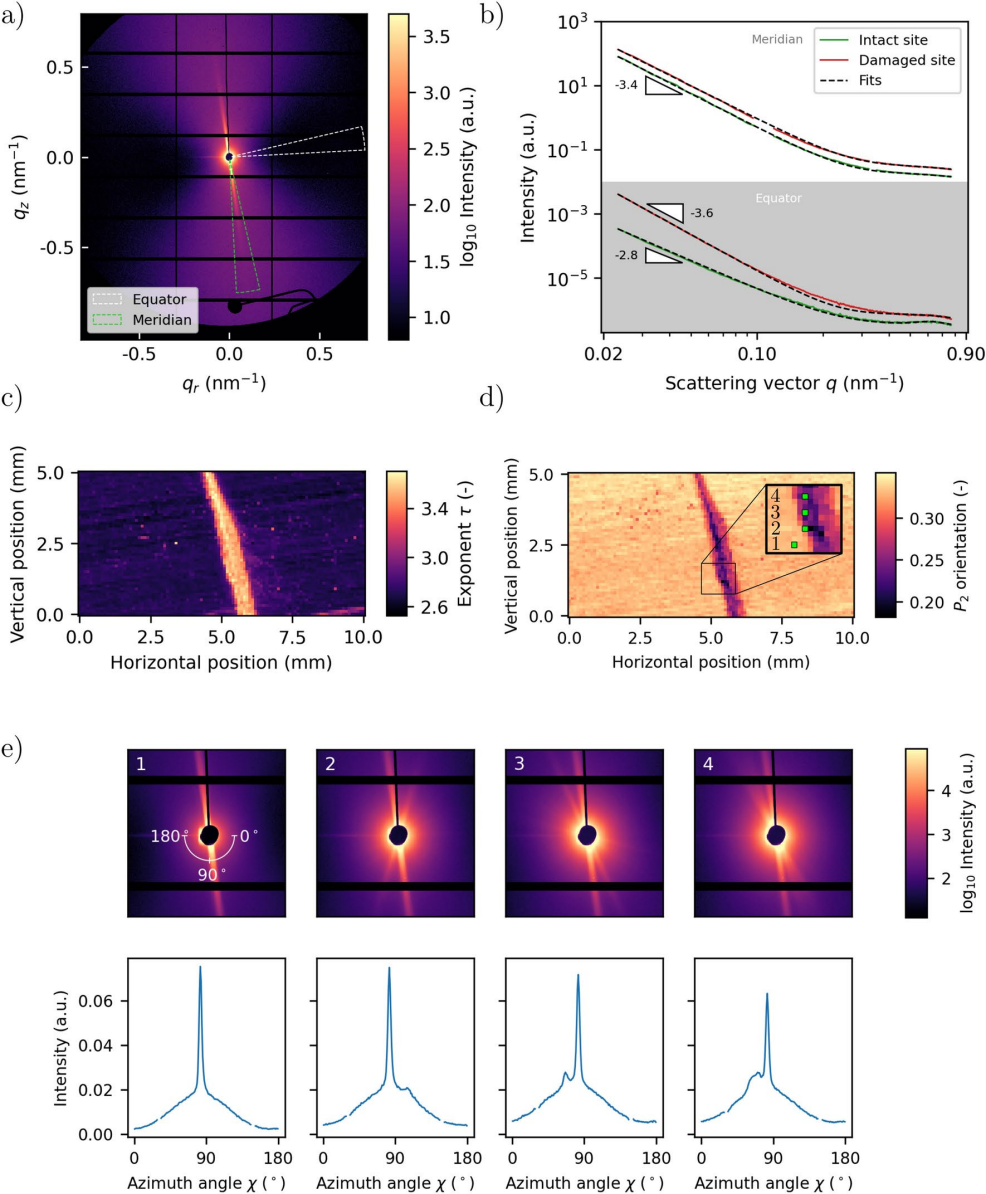
(**a**) Example of SAXS patterns. (**b**) Example of SAXS curves integrated along meridional and equatorial for intact and defect sites. (**c**) Map of the fitted exponent $\tau$. (**d**) Map of the orientation parameter $\hbox {P}_2$. e) SAXS patterns and corresponding scattering intensities integrated around azimuthal angle $\chi \in [0^{\circ },180^{\circ }]$. These correspond to the mapped positions 1-4, which are indicated by the green dots in panel d.

## Computational section

### Spatial segmentation

Following the X-ray experiments, the composite material is represented by a regular $$101\times 51$$ grid of $$100 \times 100$$$$\mu$$m squares (corresponding to the area of the SAXS/WAXS mapping), each with an associated feature vector consisting of aforementioned quantities derived from the CT and SAXS/WAXS experiments: The pseudo-density $$\hat{\rho }$$, fiber orientation coefficient $$P_2$$, power-law exponent $$\tau$$, the reduced crystallite size $$\hat{L}$$ and the distance between main $$\alpha$$-phase reflections $$\hat{q}$$. These parameters served as a basis for performing spatial segmentation, in order to help identify distinct physical and spatial domains of interest (i.e. “damaged” and “intact”). This would aid in investigating possible correlations between parameters within the respective domains, and not only across the sample as a whole.

The segmentation procedure was done using the K-means clustering algorithm^53^. This unsupervised learning technique aims to partition samples of data into a desired number of clusters *R*, by iteratively moving the centers of each cluster (in the *N*-dimensional feature space), in such a way that the within-cluster variance is minimized. I.e., for each cluster center, observations which are closer to it than any other are identified, forming the basis for updating the cluster centroid by computing the means of each feature^54^.

Due to the feature space consisting of inherently different attributes, the dataset was first standardized in order to equalize their weights during the minimization procedure. The standardization was done by independently subtracting the median value of each feature *X* (calculated using all observations) and scaling by the interquartile range (IQR), i.e.

9 $$\begin{aligned} X_{\text {scaled}} = \frac{X - \text {med}(X)}{\text {IQR}}. \end{aligned}$$

The *K*-means algorithm was then applied to the full dataset, using the Scikit-learn library.^55^ The number of clusters were based on choosing simple binary and tertiary representations of the sample, “damaged” or “intact”, with $R=2$, and the additional “intermediate” or “transition” state with $R=3$. Nevertheless, a heuristic function, the silhouette metric,^56^ was also used in order to validate the choices of *K*. For each observation in the dataset, it is calculated as

10 $$\begin{aligned} \frac{b - a}{\text {max}(a, b)}, \end{aligned}$$

where *a* is the average distance (in the *N*-dimensional feature space) of the observation to all objects in its cluster, and *b* its average distance to all objects in a different cluster. The metric ranges from 1 to -1, where 1 is the best value, 0 indicates overlapping clusters, and negative values are indicative of a sample being mis-labeled. The mean silhouette score was computed separately for values of $R=2$, 3, 4 and 5 (see Table S1 in the supplementary materials.)

### Predictive analysis

The material descriptors were investigated in terms of whether they can be used for prediction of one another. Specifically, regression analysis was performed using parameters derived from one experimental technique as a model input (features), and those from another as the output (target). For this task, the Support Vector Regression (SVR) algorithm^57^ was chosen. This method stems from the set of SVM supervised learning methods, which generally work on the principle of optimally constructing decision boundaries between classes of data that might not be linearly separable. The regression models were individually optimized and tested using different subsets of the feature vectors as both input and target variables. This resulted in two models for predicting each of the five material parameters, with the input variables being the parameters obtained from the two other experimental techniques, respectively. E.g., the pseudo-density $\hat{\rho }$ derived from CT was modeled using (1) the reduced crystallite size $\hat{L}$ and $\alpha$-phase peak distance $\hat{q}$ from WAXS, and (2) the power-law exponent $\tau$ and $P_2$ orientation from SAXS.

Due to the imbalance between the number of healthy and damaged sites on the material sample, undersampling of the majority class was performed to ensure an equal representation of both classes. The material state of each site was determined by the K-means approach, as described in the previous section. Healthy sites were randomly removed to fulfill this criterion, resulting in a reduced dataset containing 1772 samples. The data were then randomly split into 70% for training and optimizing the models, and 30% for testing their performances. Scaling of the features was done according to Eq. 9, where the population median and IQR were calculated using the training data.

The SVR models were optimized by selecting appropriate values for a set of hyper-parameters that determine the complexity of its decision function, the influence of individual training samples, and the penalization of prediction residuals during training. This process was done by performing a grid-search of a range of values for the hyper-parameters, with five-fold cross-validation on the training data. The optimized values are listed in Table S2 in the Supporting Information. The Radial Basis Function^58^ was chosen as the kernel. Finally, the models were evaluated on the external testing data, in terms of the root-mean-squared-error and $\hbox {R}^2$-score (coefficient of determination). The Scikit-learn library^55^ was used for SVR.

## Results

### CT Scans

Fig. 4 shows the results of the CT scans in terms of pseudo-density $\hat{\rho }$. The data show intricate defect compositions with macroscopic geometries largely appertaining to the fiber orientation. These include a complicated crack structure near the fractured surface and perpendicular to the fiber orientation, a large longitudinal matrix crack on the surface opposite to the impact site, as well as a band of kinked fibers.

The top-left panel of Fig. 4 shows the projected density $\hat{\rho }$ thought the (*x*, *z*)-plane of the sample. The low-density regions indicate cavities in the material (air). The less distinct but oriented intensity variations corresponds to the fiber orientation. Three sites of the sample in the top-left panel are marked by green squares, highlighting areas of interest. These are presented in the numbered panels of the figure, which show slices of the reconstructed volume. Site number 1 shows a feature perpendicular to the macroscopic fiber orientation. A band of discontinuity in the orientation of the fibers is visible, together with longitudinal splitting along the fibers within the band (elongated voids). The visibly lower density at the boundaries also indicates discontinuous and/or broken fibers. Present near the left boundaries are also multiple narrow kink bands. Site number 2 shows a near-surface (*x*, *z*)-slice with corresponding orthogonal views ((*x*, *y*) and (*y*, *z*)). This reveals chaotic crack structures permeating from top the surface and to lamina deeper in the material. Apart from the macroscopic orientation of the crack in the (*x*, *z*)-plane, the orthogonal views do not show any obvious relationship between the crack propagation and the directionality of plies and fibers in this case (i.e. little visible delamination). Site number 3 contains a view of the longitudinal feature on the sample, a long matrix crack, at the surface opposite of the impact. The orientation of the crack follows that of the fibers near the surface, but has an intricate propagation and intersection with the latitudinal crack originating from the opposite surface, as can be seen in the (*x*, *y*)-view of site number 3. The presence of kink bands (Fig. 4a, panel 1) can be connected to large compressive loads near the impacted surface^21^, and consequent tensile stresses on the opposite surface along the fiber axis, but also perpendicularly, as suggested by the longitudinal matrix crack (Fig. 4a, panel 3).

We note that the $\hat{\rho }$ projection shown in Fig. 4a has a larger sample volume and higher spatial resolution than the X-ray scattering data discussed in the following sections. A downsampled map of $\hat{\rho }$ corresponding to the area and resolution of the X-ray scattering experiments is shown in Fig. S3 (Supplementary Information).

### SAXS

Fig. 5 presents the results from the SAXS experiment. Fig. 5a shows an example of the 2D scattering pattern. The scattered pattern is a convolution of the signal from (oriented) elongated voids within the carbon fibers^29^, and isotropic scattering from the polymer matrix^28^. The equatorial and meridian directions of the scattering are indicated.

Fig. 5b shows the integrated intensity as a function of the scattering vector, along the equator (top) and meridian (bottom), from two different sites on the sample (offset in magnitude for visibility). These sites are labeled as “damaged” and “intact”, where the former originates from the cracked region, and the latter near the left edge of the map (horizontal position $\approx 0$). The scattering profiles along the meridian are characterized by power-law behaviour at low scattering angles, which levels off towards higher ones. The predominant behaviour of these intensity profiles is independent of the position of the sample (though the exponential decay is observed to increase slightly at some sites near the damage, see Fig. S4 in the Supplementary Information).

**Fig. 6:**
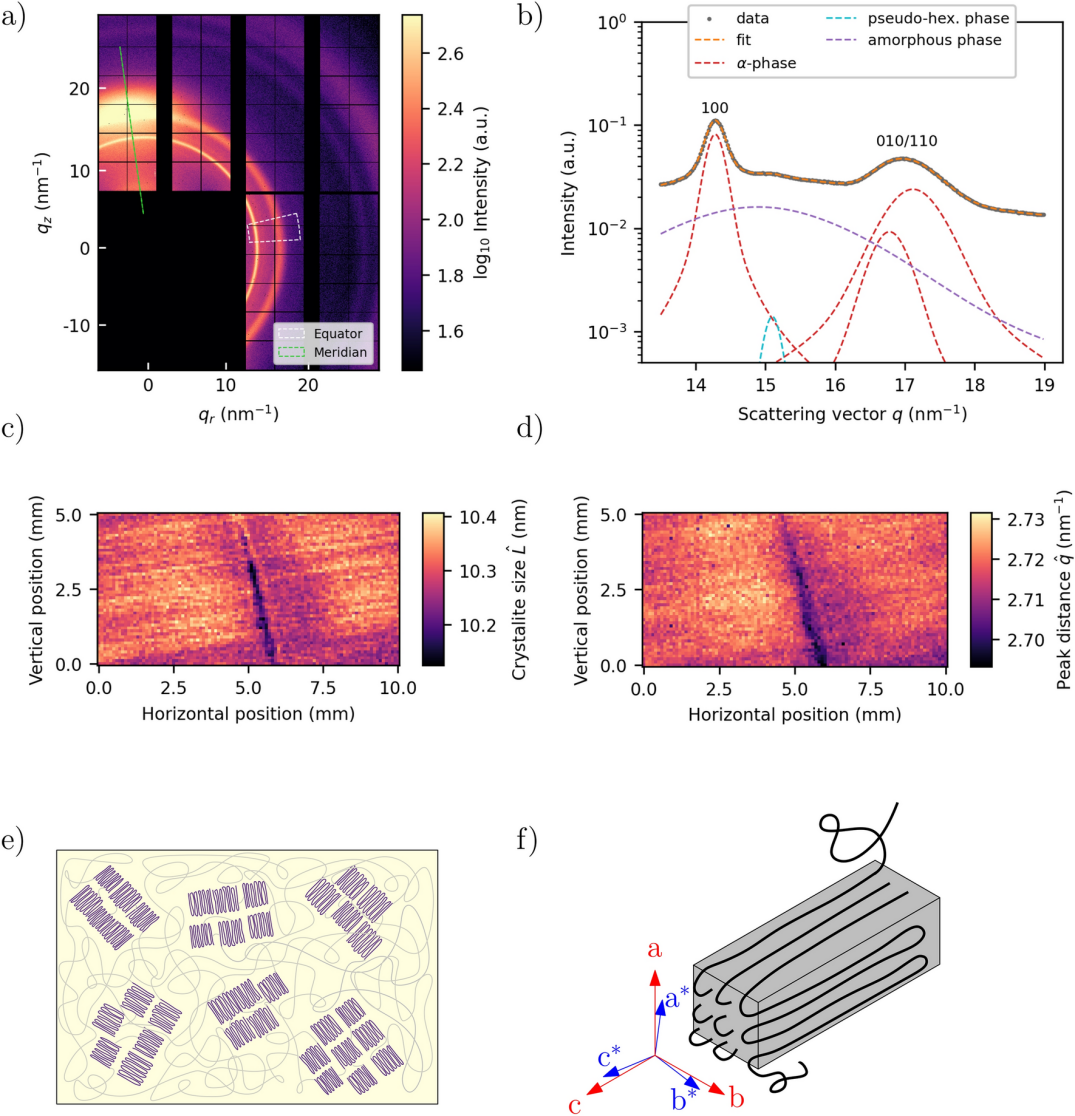
(**a**) Example of WAXS patterns. (**b**) Example of WAXS curve integrated along the equatorial. (**c**) Map of crystal size. (**d**) Map of distance between 100 and 010/110 reflections. (**e**) Illustration of crystallites within amorphous polymer. (**f**) Chain-folded polyamide-4,10 and the unit cell vectors. The polymer chains are oriented along the *c* direction, and the inter-sheet and inter-chain directions correspond to the *a* and *b* axes, respectively.

The integrated intensity of the equator is shown in the bottom-half of Fig. 5b. Although it shows a similar characteristic to that of the meridian, the initial slope of the scattering is more shallow, with the addition of a weak correlation peak at the high end of the scattering angles. The correlation peak is isotropic, and therefore also present at the meridian angle, though rather obscure due to the orders of magnitude intensity difference between the meridian and equator. The correlation peak is characteristic for the periodic organization of crystallites within the amorphous matrix^59^, but is also consistent with the measured crystallite sizes (see Fig. 6c below.) Contrary to the intensity profiles of the meridian region, the behaviour in the low *q* regions of the equator varies more greatly depending on whether or not it originates from a site near the impact. At an intact site, the intensity decays exponentially with a power-law exponent $$\tau = 2.6$$, increasing to $$\tau =3.6$$ at damaged sites. This is further exemplified in Figure 5c, showing the fitted values of $$\tau$$ mapped onto each position on the sample. Generally, the macroscopic crack structure perpendicular to the fiber orientation (Fig. 4a, panel 2) is dominant, but the longitudinal crack (Fig. 4a, panel 3) is also visible as slightly increased values of $$\tau$$. The power-law behaviour of the intensity can be connected to two distinct self-similar object, where an exponent $$\tau < 3$$ corresponds to a mass fractal, and $$3< \tau < 4$$ a surface fractal, with respective fractal dimensions $$D_{\text {mass}} = \tau$$ and $$D_{\text {surface}} = 6 - \tau$$^60^. The power-law behaviour of the intensity is evident in the range $$0.02< q < 0.1$$$$\hbox {nm}^{-1}$$, amounting to scattering from objects between approx. 60 and 300 nm in size.

The intensity of the correlation peak at approx. $$q=0.6$$$$\hbox {nm}^{-1}$$ is comparatively weak at many of the damaged sites, and unique maxima are problematic to determine. This leads to ambiguous parameters when fitting these profiles (see Fig. S5 in the Supplementary Information).

Fig. 5e shows four examples of the low-*q* 2D scattering from different sites on the sample, with the corresponding radially integrated intensity curves. The left-most panel exemplifies the scattering from an intact site, while panels 2, 3 and 4 are from sites within the cracked region (sites annotated in Fig. 5d). The figure shows a single intensity maximum (in each hemisphere) for the intact site, consistent with a high degree of alignment of the fibers^24^. Sites 2, 3 and 4 show scattering from the cracked region. There is at least one extra maximum, indicating multi-modal fiber orientation distributions. This signifies a region in the sample where the fibers are kinked or misaligned (either locally or on average in the illuminated volume), with the intensity magnitude of the streak maxima proportional to the amount of misaligned fibers in the respective structures. This observation is consistent with the observations from the CT scans in Fig. 4, pointing out the bands of misaligned fibers. 

Fig. 5d shows the fiber alignment orientation parameter $P_2$ mapped onto the sample (Eq. 4). This parameter also displays a distinct change in behaviour in the cracked region of the sample. It drops from approx. 0.35 in the healthy region to 0.25 in the crack vicinity, indicating less anisotropic scattering with respect to the meridian reference angle, i.e. multiple and/or wider intensity maxima (Fig. 5e). Furthermore, there is a presence of a gradient of the $P_2$ parameter map. Comparing it to the CT scan (Fig. 4 panel 2), the gradient looks to be associated with the geometry of the crack, with the heaviest damage in proximity to the sample surface (left boundary of $P_2$ feature in Fig. 5d), decreasing with depth (right boundary in $P_2$ map).

### WAXS

Fig. 6 illustrates the results from the WAXS experiment. Fig. 6a shows a typical diffraction pattern where diffraction from polyamide-4,10 is represented by the two innermost rings^50^. The anisotropic component along the meridian corresponds to the [020] reflection of the fiber graphite^25^. Fig. 6b shows an example of the azimuthally integrated intensity in the equatorial wedge of the diffraction pattern, accompanied by a representative curve fit to the profile. The two distinct Bragg peaks are reflections of the [100] and [010/110]-planes of the PA-4,10 $\alpha$-phase unit cell, the halo corresponds to the amorphous polymer phase, and the slight shoulder of the [100] peak is from a pseudo-hexagonal phase^50^.

Fig. 6c and d display maps of the estimated crystal size $\hat{L}$ (Eq. 8) and the inter-peak distance of the 100 and 010/110 reflections $\hat{q}$ (Eq. 6), derived from the resulting parameters of the curve fits to the WAXS profiles (akin to Fig. 6b.) The corresponding maps of parameters from the individual peaks are shown in Fig. S6 (Supporting Information). The crystallites within amorphous polymer and the polyamide $\alpha$-phase unit cell are illustrated schematically in Fig. 6e and f. The apparent size of the crystallites decreases slightly when approaching the damaged region horizontally from the right, with an abrupt drop near the middle. A similar gradient is visible in the map of the peak distance $\hat{q}$, though it is more gradual across the width of the cracked region. Both parameters $\hat{L}$ and $\hat{q}$ also look to be slightly altered in proximity to the longitudinal crack structure along the fiber orientation (Fig. 4 panel 3). Changes to the value of $\hat{q}$, i.e. alterations of the $\alpha$-phase unit cell lengths, can be an indication of frustration, and a smaller value of it is associated with lattice defects in similar polyamides^61^.

### Multi-scale correlation

**Fig. 7:**
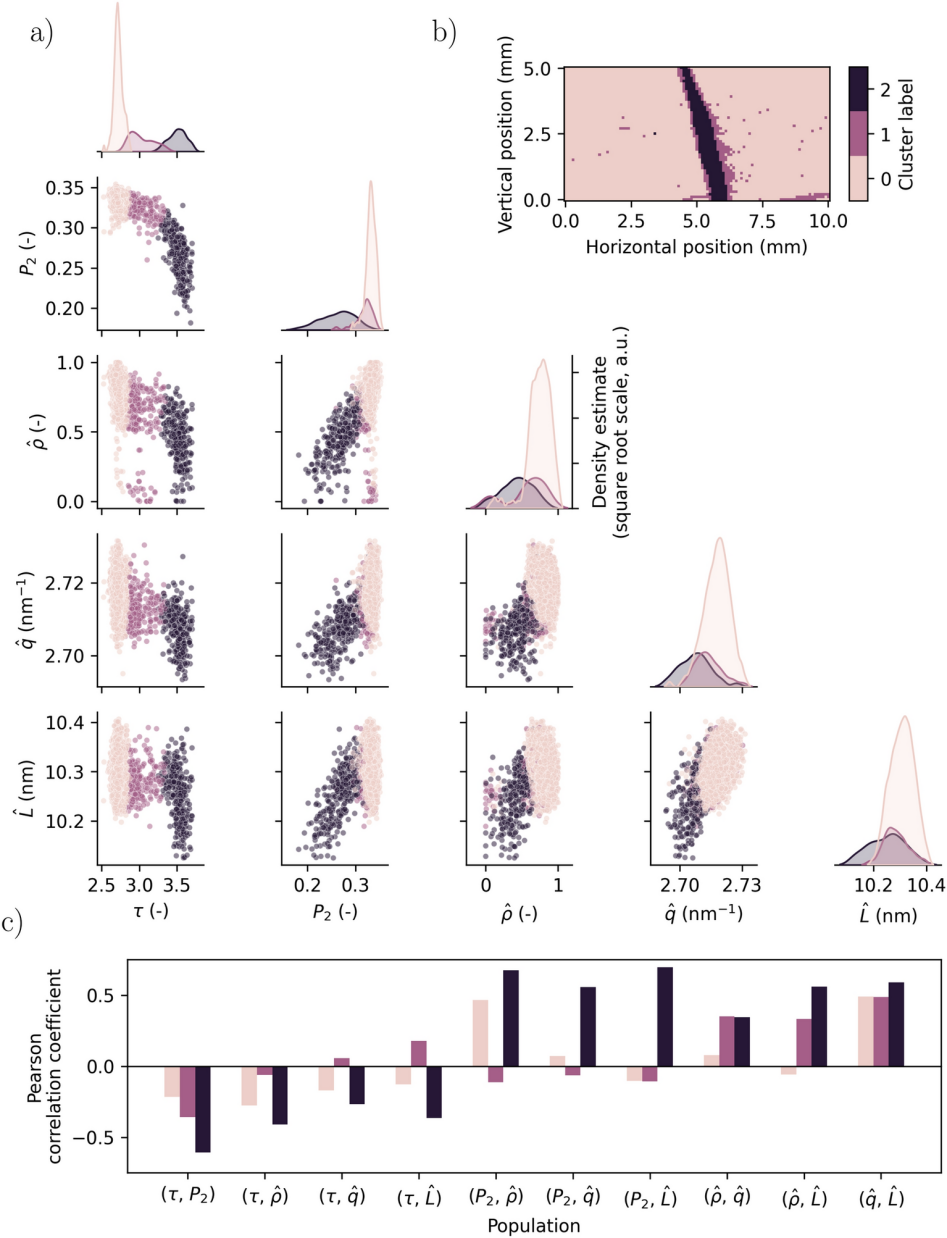
Results from the spatial segmentation using parameters derived from the various experiments: The pseudo-density ($\hat{\rho }$), orientation parameter ($P_2$), power-law exponent ($\tau$), the distance between main $\alpha$-phase reflections ($\hat{q}$) and the reduced crystallite size ($\hat{L}$). (**a**) Pairwise parameter relationships, represented by a grid of axes showing each parameters’ numerical value as a function of another’s. The columns and rows of the grid share *x* and *y* axes, respectively. The data points are colored according to the assigned cluster label (see **b**). The diagonals are density estimates (square root scale) of the column representative’s marginal distribution divided into the respective clusters. (**b**) The assigned cluster labels the mapped onto the sample sites. (**c**) Pairwise Pearson correlation coefficients calculated separately for the three clusters.

Fig. 7 illustrates the domain segmentation with $R=3$ clusters and correlations between the identified parameters. Fig. S7 shows the corresponding figure with $R=2$ clusters (Supporting Information). For comparison, Fig. S8 lists the correlation coefficients between the parameters across the whole sample (without partitioning them into clusters).

Fig. 7a shows relationships between the parameters in a pair-plot correlation matrix, in which the numerical values of pairs are plotted against each other (created using Seaborn^62^). The marginal distributions of the parameters are also shown along the diagonal of the matrix. The colors represent the cluster label assigned by the K-means algorithm, and the distributions of the parameters are shown for the populations from each of these (square root scale, for visibility). Fig. 7b shows the K-means’ assigned cluster labels of all of the observations, mapped onto each of the sites of the sample. The label “0” corresponds to a “healthy” domain of the sample, and the label “2” as a “damaged” domain. The third domain, with a label of “1” is present at the boundaries of the damaged region, and is regarded as a “transition” domain. This map is thus a low-dimensional representation of the sample, composed of all the discussed parameters of the various length scales. The mapped labels show distinctly the features also seen in the CT, SAXS and WAXS data (Figures 4, 5 and 6 respectively), but qualitatively it is more resemblant of the mapped SAXS parameters, $\tau$ and $P_2$ (Fig. 5c,d). (See also Fig. S3 for corresponding map of the pseudo-density $\hat{\rho }$).

Considering the power-law exponent $\tau$ and its distributions in the three clusters (Fig. 7a), they are mostly non-overlapping and can clearly be distinguished. To a lesser degree, this also applies to the $P_2$ parameter, though the sites in the transition domain share values with those in the healthy domain. The parameters derived from the WAXS data have predominantly overlapping distributions w.r.t. the labels, with only one of the tails of the transition domain distribution separate from the rest. The parameter distribution of the pseudo-density $\hat{\rho }$ are quite distinctive in the healthy and damaged domains, but for cluster 1 it is bimodal, i.e. the transition domain contains both high and low material density regions. The sites of the transition domain contributing to the low density counts in this distribution, are mostly those near the longitudinal crack, which is clearly a region of the sample with detrimental damage (see e.g. Fig. 4 or Fig. S3 in the supplementary material).

From the the pair-plots themselves (Fig. 7a), distinctive differences in behaviour within the respective domains can be seen. Generally, the parameters are to a lesser degree correlated in the healthy domain, but relationships between them become visible when approaching the damaged domain. Figure 7c characterizes this observation, by quantifying linear correlations between parameter-pairs in the respective domains, using the Pearson correlation coefficient^63^. There is also a tendency of higher correlation between parameters stemming from the same experiments, in all of the three domains (e.g. SAXS parameters are more correlated to each other than SAXS and WAXS parameters). The exception to this is the ($P_2$, $\hat{\rho }$)-pair, showing a strong correlation in the damaged domain. This might be because both $P_2$ and $\hat{\rho }$ are associated with the same macroscopic structure.

**Fig. 8:**
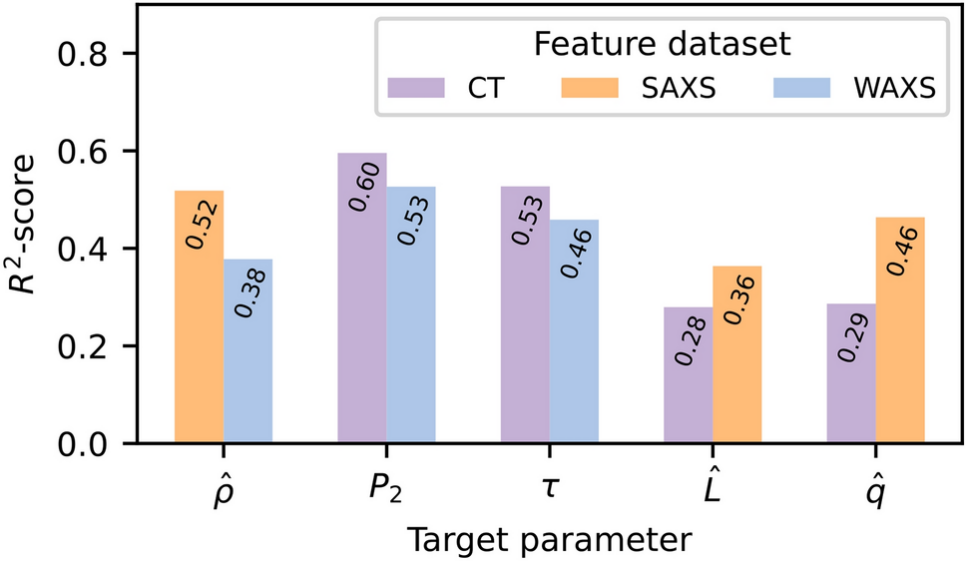
$\hbox {R}^2$-scores on the test data of the SVR models optimized for predicting the five material parameters. The color legend shows which dataset (parameters derived from the respective experimental techniques) is used in the training and optimization process.

Fig. 8 shows the results of the regression models for predicting the respective material parameters using the SVR algorithm, in terms of the $\hbox {R}^2$-score on the test data. The root-mean-squared-errors of the models are listed in Table S2 in the Supplementary Material. The bars in Fig. 8 represent two models for predicting each of the five material parameters, using feature variables stemming from the different experimental techniques (i.e., CT, SAXS, WAXS). The models optimized for predicting parameters derived from the SAXS, namely the $P_2$ orientation and power-law exponent $\tau$, perform the best, achieving an $\hbox {R}^2$-score of up to 0.60. This is not an unexpected result considering the high correlation (in absolute value) between the $P_2$ orientation and other parameters in the damaged domain (cf., Fig. 7). Using the pseudo-density $\hat{\rho }$ as a single input parameter, the performance is slightly higher than with the two WAXS parameters ($\hat{q}$ and $\hat{L}$), suggesting that this single parameter explains more of the variance in the SAXS data than the two WAXS parameters combined. The pseudo-density $\hat{\rho }$ can also be reliably be predicted from the SAXS features. Conversely, the modelling of the crystallite size $\hat{L}$ and peak distance $\hat{q}$ results in poorer performances, especially with the pseudo-density as the only predictor. This can be understood from considering the gradients present in the WAXS parameters in the healthy domain (Fig. 6 c and d), which are not present/much less distinct in both the SAXS and CT feature sets. Additionally, the pair plots in Fig. 7a show that the WAXS parameters $\hat{q}$ and $\hat{L}$ have large variances in the healthy domain, while the other parameters are approx. constant. This indicates that there is little information in the CT and SAXS datasets to account for the variance in $\hat{q}$ and $\hat{L}$.

## Discussion

We chose the K-means algorithm based on its apparent simplicity, and show that this algorithm can be applied for investigating sample sites that might not be segmented manually. This approach does not strictly classify the structural state of the sites, but works as a tool for identifying areas where potentially secluded relationships between parameters relating to different aspects of the material can be investigated. The number of clusters was chosen to reflect binary and tertiary views of the sample’s state, i.e. domains. Two domains were found experimentally to be either clearly intact or clearly damaged. The third state was interpreted as an transition state in between these two states. The cluster representing damaged state can also be viewed as a region where predominantly all parameters of the respective sites have altered values as compared to the intact state, whereas the transition state is one where only a subset of the parameters are altered (e.g., the $\tau$ parameter). As the objective of the K-means algorithm is to minimize the distance between *all* the points in respective clusters, distinguishing between these two cases might be difficult without either considering the underlying data, or increasing the complexity of the model (e.g. increasing the number of clusters). We can also identify gradients across the states (e.g., $\hat{q}$ and $\hat{L}$ in the WAXS). The physical sites representing distinct states are not scattered across the sample, but form spatially well defined “domains”, where the intact domains are separated from a damaged domain by transition domains. This provides us with a phenomenological justification to consider three clusters.

The variance of the respective parameters measured across the whole of the material sample can be a large contributor in determining what constitutes a cluster (when using Euclidian distance as a metric). This is exemplified by looking at the distinctiveness of the $\tau$-distributions w.r.t. the cluster labels (Fig. 7a), and comparing the ranges in the parameter spaces for $\tau$ and $\hat{q}$. Incidentally, the former parameter is arguably a stronger indicator of damage than the latter, though this correlation between “healthy-damaged-variance” does not always need to be the case. We note that $\hat{\rho }$ from the CT experiment is the only parameter directly representing truly macroscopic (> $\mu$m) scale, whereas there are two each for the smaller scales, the latter thus having a greater individual influence in determining the cluster means.

Predictive analysis was done using the Support Vector Regression with the radial basis function as the kernel, where features representing one length scale were modelled as predictors for parameters representing another. The results suggest that each of the presented experimental techniques can be used individually to give indications of changes to the material on both the 1 $\mu$m to 1 mm and 30 to 300 nm scales. Though, not unexpectedly, the nanoscopic (WAXS) material features could not be reliably predicted by larger length features. For example, the $P_2$ orientation can be predicted using the crystal size $\hat{L}$ and inter-peak distance $\hat{q}$ as features, but this property does not seem commutative, i.e. $P_2$ cannot predict $\hat{q}$. The performance of the models suggest that the identified material parameters can be used as regressors, though there is generally a high variance between the parameter-pairs (Fig. 7), which is challenging to model using the present dataset alone. Similarly, the parameters may also behave differently in relation to each other in the respective domains, exemplified by e.g. $\hat{\rho }$ vs. $\tau$ in Fig. 7a. This suggests that additional descriptive features, rather than higher complexity models might be needed in order to further improve predictive performance.

The $P_2$ orientation parameter is commonly used for estimating fiber orientation distributions from X-ray data^24,25^ but is proportional not only to the magnitude of the misalignment but also to the number of misaligned fibers. Thus its physical interpretation should be taken with care in cases of bimodal (or multi-modal) X-ray intensity distributions over the azimuth, and higher order $P_n$ coefficients might be better fitted for discriminating such behaviour^64^. Concerning the fiber orientation in the described sample, the mapped $P_2$ orientation parameter shows a close correlation to the pseudo-density $\hat{\rho }$, with a wide distribution and a visible gradient in the damaged region (Fig. 5d and Fig. 7a). This is consistent with the presence of voids in kink band-type defects, both here and elsewhere.^21^ There is also a clear connection between $P_2$ and the visually apparent fiber misalignment seen in the CT scans (Fig. 4). We thus find that $P_2$ serves as a simple and applicable parameter for identifying kink bands. However, it is ambiguous in terms of whether there is a large local contribution or multiple smaller contributions from inherently different structures in the material, in which further parametrization of the CT data could conceivably clarify. For example, segmentation efforts could quantify the (local macroscopic) fiber alignment and void content^17,19^, as well as various crack structures^18^, from which additional pixelwise descriptors also could be computed and compared to the nanoscopic material descriptors.

The SAXS data along the equatorial shows a power-law behaviour which is altered predominantly in the region near the crack transverse to the fiber direction, but also slightly along the longitudinal matrix crack visible in the CT scans (Fig. 4). The intensity curves can be connected to a fractal hierarchy of scattering object up to 300 nm, characterized by a mass fractal behavior in the intact domain and a surface fractal behavior near the fracture. We expect that the highest cut-off point for the fractal size is beyond the present observation window and would require a lower *q*-range to acquire^60^. These results should be compared to the studies of similar polyamides where this behavior is attributed to the scattering from objects pertaining to nano sized cavities, and where the cut-off point is found in the low $\mu$m-range^28^.

Though we attempt to exclude scattering contribution from the fibers by measuring the power-law exponent perpendicular to the azimuthal maximum, the fiber misalignment in the cracked region affects the scattered intensity with respect to the azimuthal angle. So for the data presented in Fig. 5b and c, we are not able to fully exclude the fiber kink-signal contributing to the intensity along the equator, i.e. the equatorial intensity can stem from two different structures. This effect might be minimized further by integrating along the azimuth angle of lowest intensity, but in the general case, an elaborate 2D fitting procedure might be needed to fully deconvolute scattering from the respective structures.

PA-4,10 is a typical semi-crystalline polymer, and we identify an interference maximum at about 0.6 $\hbox {nm}^{-1}$, with contributions from both crystal size and crystal-crystal (or crystal-amorphous) periodicities. This becomes more prominent in the equatorial rather than meridional direction, and for intact rather than damaged areas. This maximum is interpreted in terms of various detailed models including those described in Refs^65,66^. However, the scattering from these polymer structures are comparatively weak in the domain of severe damage, and obtaining sufficient fits for such parametrizations, and even simple Lorentzians, is problematic (see Fig. 5b and S5 in the Supporting Information). This is not due to insufficient *q*-range or poor signal-to-noise ratio but simply illustrates a limitation of the method.

In addition to (true) crystal size affecting the peak width of the Bragg reflections, micro-strains and dislocations in the crystal lattice may also contribute^31,52^. Though, without higher order reflections, the respective contributions cannot be deconvoluted. We note that the crystallite size estimated from the observed SAXS maximum does agree with that determined from the WAXS reflections, $\sim 10.4$ nm, suggesting that the crystal size accounts for most of the peak broadening. The observed peak broadening is unlikely the result of opposing effects, i.e. the peak will broaden both with decreasing crystal size and with emerging micro-strains, both of which are plausible scenarios in a region of structural defects. Moreover, if strain contribution was significant, this would not alter correlations between parameters or found predictive analysis, but simply modify the physical interpretation of the reduced $\hat{L}$ parameter.

PA-4,10 has several polymorphs and they differ slightly from those of the most common PA-6,6 and other nylons^50^. Since the dominant alpha-phase accounts most of polymer properties, we have selected this for our considerations. Measurements of the polyamide $\alpha$-phase unit cell show that the inverse space distance between the 100 and 010/110 reflections ($\hat{q}$) increases when approaching the fractured region. The gradient of $\hat{q}$ reaches larger distances away from the crack, compared to those of the other discussed parameters (also in the regions near the longitudinal matrix crack that is located on the other side of the sample in Fig. 4a panel 3, and Fig. 6d.) From other reports of morphological transformations of polyamides during heating and cooling^59,67^, together with pronounced heat dissipation during failure events in composites^5^, we suggest that the altered values of the reduced peak distance $\hat{q}$ and crystallite size $\hat{L}$ can be connected to such a phenomenon, though it is difficult to conclude from the present datasets alone.

## Conclusion

We show how information from the X-ray datasets can be used to parameterize structural information over a number of length scales with correlations to larger defects, and a looser correlation with microstructural data relating to crystal size and strain. Furthermore, these parameters were used as input-output pairs in a regression analysis, demonstrating that relationships between the material descriptors can be modelled. We also note that the two-layer (crystal-amorphous) periodicity — typically used to characterize semi-crystalline polymers — cannot be easily employed in this parametrization. The results may be useful in cases where one does not have easy access to comprehensive X-ray datasets, including product lines or field conditions, or when bulky material blocks are considered. They may also serve as a starting point for developing physical models connecting the observed phenomena.

Forthcoming research should expand these ideas to more complicated composites, such as [$0{^\circ }/90{^\circ }$] cross-ply lay-ups and other configurations. Other algorithms for data clustering should also be tested, for example Gaussian mixture models, which takes into account features of unequal variances. A more nuanced segmentation effort with additional clusters could be attempted alongside a larger number of samples and datasets. Additional parameters could also be defined, including ones that are more descriptive of the average/local fiber orientation and microstrain.

## Supplementary Information

Supplementary Information.

## Data Availability

The datasets used and analysed during the current study is available from the corresponding author on reasonable request.
