# Supplementary material for: Multi-scale correlation of impact-induced defects in carbon fiber composites using X-ray scattering and machine learning
Source: Sci Rep. 2024 Oct 17;14:24393. doi: 10.1038/s41598-024-76105-6 (PMC11487195; doi:10.1038/s41598-024-76105-6)
Supplement: Supplementary file 1 — Supplementary Information. [file 41598_2024_76105_MOESM1_ESM.pdf]

Supplementary Information

Multi-scale correlation of impact-induced defects in carbon fiber composites using X-ray scattering and machine learning

*Alexander H. Sexton**, *Heikki Suhonen*, *Mathias K. Huss-Hansen*, *Hanna Demchenko*, *Jakob Kjelstrup-Hansen*, *Matthias Schwartzkopf* and *Matti Knaapila**

A. H. Sexton, H. Demchenko, M. Knaapila
Department of Physics
Norwegian University of Science and Technology
7491 Trondheim, Norway
E-mail: alexander.h.sexton@ntnu.no, matti.knaapila@ntnu.no

A. H. Sexton, M. Knaapila
DPI, P.O. Box 902, 5600 AX Eindhoven, The Netherlands

H. Suhonen
Department of Physics, University of Helsinki, 00014 Helsinki, Finland

M. K. Huss-Hansen, J. Kjelstrup-Hansen
NanoSYD, Mads Clausen Institute, University of Southern Denmark, 6400 Sønderborg, Denmark

J. Kjelstrup-Hansen
SDU Climate Cluster, University of Southern Denmark, 5230 Odense, Denmark

M. Schwartzkopf
Deutsch Elektronen Synchrotron DESY, 22607 Hamburg, Germany

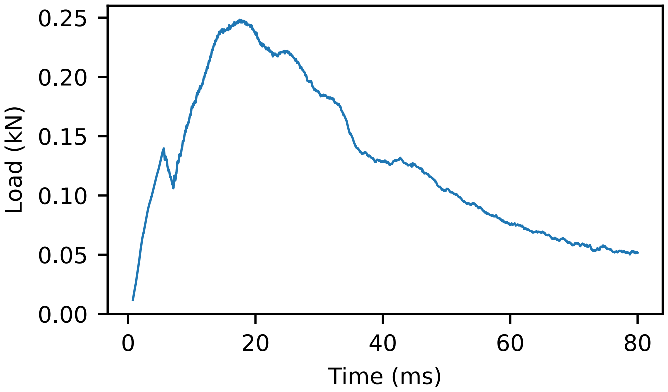

Figure S1: Force-time history for the impacted sample with the impact energy of 4 J.

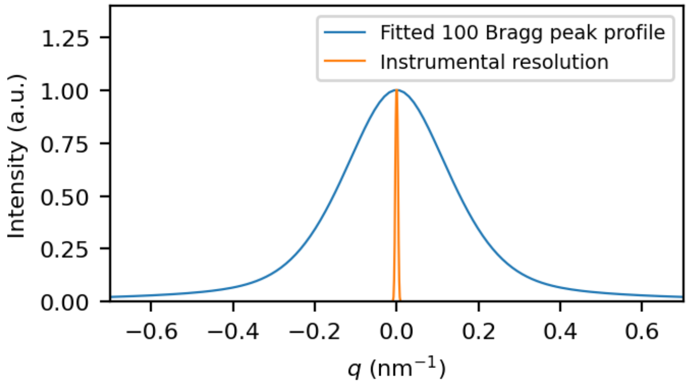

Figure S2: Radial profile of the fit to the 100 Bragg reflection together with the instrumental resolution. The peaks are zero centered for comparison.

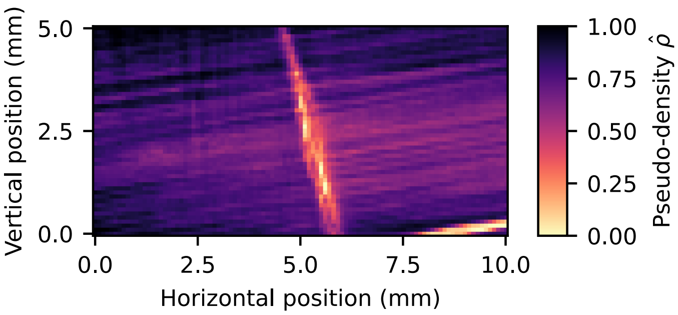

Figure S3: Downscaled CT projection used for correlating with parameters from SAXS/WAXS data sets.

Fig. S1 plots the force-time curve for the discussed impact. Fig. S2 shows the profile of the fitted 100 reflection together with the direct X-ray beam for comparison. A fit to the direct beam profile gives an estimated FWHM of the instrumental function of 0.0069 nm^{-1}. Further contributions of peak broadening due to instrumentation comes from the sample thickness itself,[S1] which at 2 mm gives a maximum relative deviation in the scattering vector q of $\Delta q/q = 0.017$ (at $q = 16$ nm^{-1} and a sample-detector-distance of 225 mm.) As the thickness variation across the sample is less than 90 μm, the effect on the peak width from this variation becomes insignificant. Fig. S3 shows the map of pseudo-densities for the spatial resolution and area that correspond to the X-ray scattering maps (see the orange dashed lines in Fig. 2).

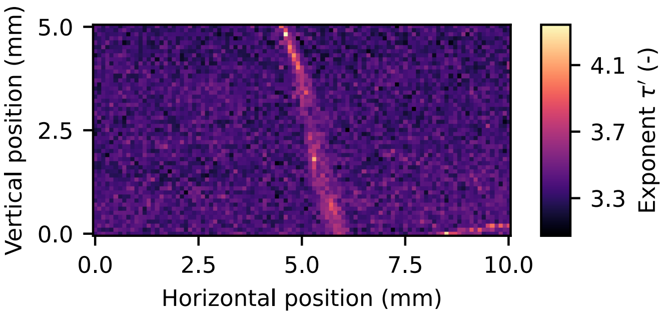

Figure S4: Map of the fitted power-law exponent of the meridian intensity profile (cf., Fig. 5b).

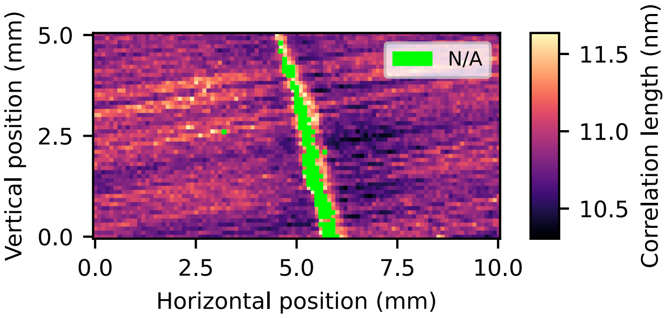

Figure S5: Map of correlation lengths deduced from the SAXS profile equatorials (cf., Fig. 5b). Pixels marked in green indicate an ambiguous result from the fitting procedure.

Fig. S4 shows the power-law exponents fitted to the SAXS data along meridional. Fig. S5 shows the correlation lengths deduced from the SAXS data along the equatorial. Examples of the fits mapped for Fig. S4 and S5 are shown in Fig. 5b.

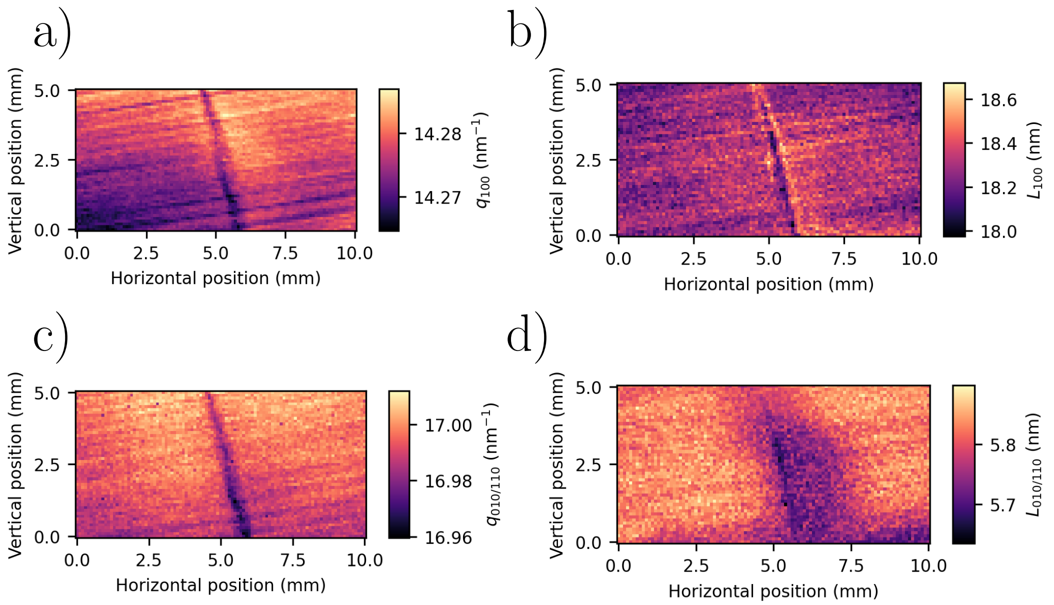

Figure S6: Maps of individual peak parameters from fits to the α-phase Bragg reflections (cf., Fig. 6b).

Fig. S6 shows the peak positions and crystal sizes as estimated from the fits to the main reflections. An example of these fits are shown in Fig. 6b.

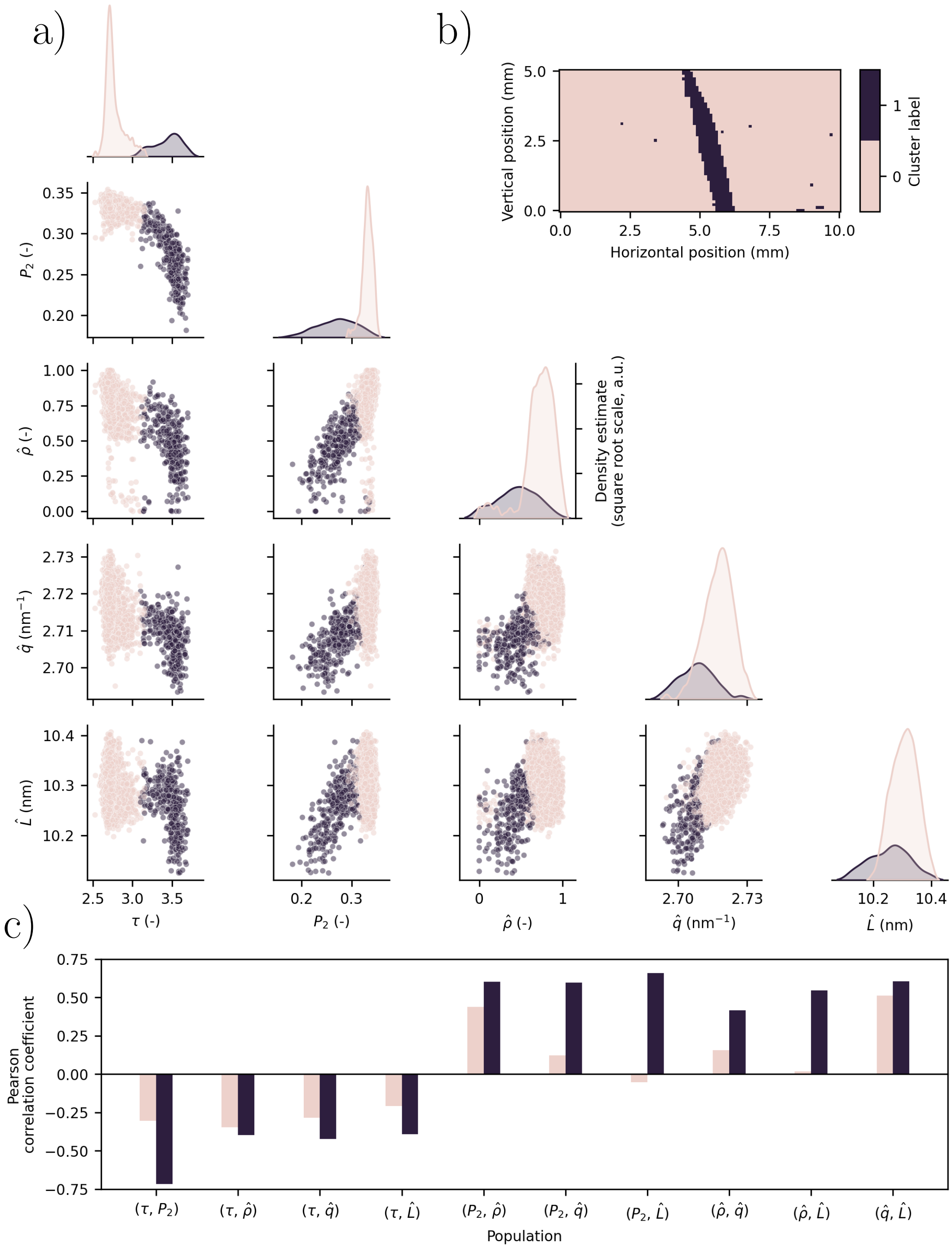

Figure S7: a) The pairwise parameter relationships for the identified parameters: The pseudo-density ($\hat{\rho}$), orientation parameter (P_2), power-law exponent τ, the distance between main α-phase reflections ($\hat{q}$) and the reduced crystallite size ($\hat{L}$). The y-values are shared across a single row (representing one parameter) and the x-values (representing another) are shared across a single column. The data points are colored according to the assigned cluster label (see b). The diagonal plots are density estimates (square root scale) of the column representative's marginal distribution divided into the respective clusters. b) The assigned cluster labels the mapped onto the sample sites. c) Pairwise Pearson correlation coefficients for each parameter pair and cluster.

Fig. S7 compiles the results from the spatial segmentation for all the parameters with two spatial clusters (cf., Fig. 7 with three clusters).

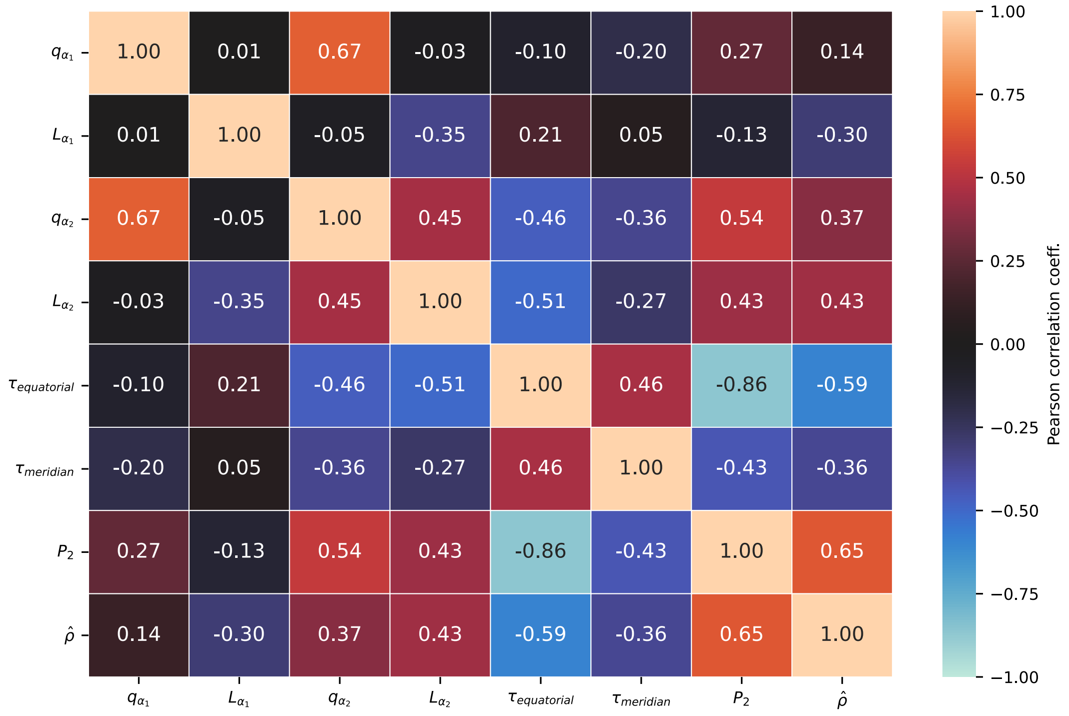

Figure S8: The correlation matrix with all the parameters across the sample.

Table S1: Mean silhouette scores using the K-means clustering algorithm for $K = 2, 3, 4$ and 5.

Number of clusters K	Mean silhouette score
2	0.88
3	0.74
4	0.59
5	0.36

Fig. S8 shows the correlation matrix for all the non-reduced parameters across the studied sample area (cf., Fig. 2). Note that the parameters shown here are not reduced and are given as q and L and not as $\hat{q}$ and $\hat{L}$, for example. Table S1 lists the mean silhouette scores for studied cluster numbers. Table S2 lists the SVR models' tuned hyper-parameters found by a grid search, and the performances on the training and test data in terms of the root-mean-squared-error (RMSE) and R^2-score. The parameter space used in the grid search was $C \in [0.01, 0.1, 1, 10]$, $\gamma \in [0.0001, 0.001, 0.01, 0.1]$ and $\varepsilon \in [0.001, 0.01, 0.1, 1]$.

Table S2: Tuned hyper-parameters and performance of the SVR models.

Target variable	Feature variables	Train *RMSE*	Test *RMSE*	Train R^2	Test R^2	P1 - Regularization strength C	P2 - Decision boundary curvature γ	P3 - Penalty tube-width ε
$\hat{\rho}$	τ, P_2	0.13	0.12	0.47	0.52	1.0	0.1	0.1
	$\hat{q}$, $\hat{L}$	0.14	0.14	0.39	0.38	10	0.1	1.0
P_2	$\hat{\rho}$	0.018	0.017	0.57	0.60	10	0.1	1.0
	$\hat{q}$, $\hat{L}$	0.018	0.018	0.57	0.53	10	0.1	1.0
τ	$\hat{\rho}$	0.21	0.21	0.49	0.53	10	0.1	0.1
	$\hat{q}$, $\hat{L}$	0.23	0.23	0.38	0.46	10	0.1	1.0
$\hat{L}$	$\hat{\rho}$	0.038 nm	0.038 nm	0.22	0.28	10	0.1	1.0
	τ, P_2	0.035 nm	0.034 nm	0.34	0.36	1.0	0.1	0.01
$\hat{q}$	$\hat{\rho}$	0.0050 nm^{-1}	0.0053 nm^{-1}	0.35	0.29	10	0.1	0.001
	τ, P_2	0.0045 nm^{-1}	0.0046 nm^{-1}	0.47	0.46	1.0	0.1	0.01

References

[S1] Pauw, B. R. Everything saxs: small-angle scattering pattern collection and correction. *J. Phys.: Condens. Mater.* **25**, 383201. doi:10.1088/0953-8984/26/23/239501 (2013).
